# A pilot study to assess use of fluorescent lotion in patient care simulations to illustrate pathogen dissemination and train personnel in correct use of personal protective equipment

**DOI:** 10.1186/s13756-016-0141-4

**Published:** 2016-10-20

**Authors:** Heba Alhmidi, Sreelatha Koganti, Myreen E. Tomas, Jennifer L. Cadnum, Annette Jencson, Curtis J. Donskey

**Affiliations:** 1 Research Service, Louis Stokes Cleveland VA Medical Center, Cleveland, OH USA; 2 Geriatric Research, Education, and Clinical Center, Cleveland Veterans Affairs Medical Center, 10701 East Boulevard, Cleveland, OH 44106 USA; 3 Case Western Reserve University School of Medicine, Cleveland, OH USA

**Keywords:** Bacteriophage MS2, Fluorescent marker, Personal protective equipment, Healthcare personnel, Training

## Abstract

**Background:**

Simulations using fluorescent tracers can be useful in understanding the spread of pathogens and in devising effective infection control strategies.

**Methods:**

During simulated patient care interactions in which providers wore gloves and gowns, we evaluated environmental and personnel dissemination of fluorescent lotion and bacteriophage MS2 from a contaminated mannequin. The frequency of skin and clothing contamination after removal of personal protective equipment (PPE) was compared before versus after an intervention that included education and practice in PPE donning and doffing.

**Results:**

Ten healthcare personnel participated in 30 pre-intervention and 30 post-intervention patient care simulations. Fluorescent lotion and bacteriophage MS2 were rapidly disseminated to touched surfaces throughout the room; there was no difference in the frequency of contamination before versus after the PPE training intervention. After the intervention, there was a decrease in skin and/or clothing contamination with fluorescent lotion (9/30, 30 % versus 1/30, 3 %; *P* = 0.01) and bacteriophage MS2 (8/30, 27 % versus 2/30, 7 %; *P* = 0.08) and there was a significant reduction in the concentration of bacteriophage MS2 recovered from hands (0.31 versus 0.07 log_10_plaque-forming units; *P* < 0.01).

**Conclusions:**

Our findings suggest that simulations with fluorescent lotion can be a useful teaching tool to illustrate the spread of pathogens and provide further evidence that simple PPE training interventions can be effective in reducing contamination of personnel.

## Background

Transmission of healthcare-associated pathogens occurs through interplay between patients, healthcare personnel and the environment. Patients colonized or infected with pathogens shed organisms onto their skin, clothing, bedding, and nearby environmental surfaces [1–4]. The hands of personnel serve as the major vector for transmission of pathogens [3–6]. Susceptible patients may also acquire pathogens through direct contact with contaminated surfaces or portable equipment [1–3]. The environment is considered an important source for transmission of several pathogens, including *Clostridium difficile*, methicillin-resistant *Staphylococcus aureus* (MRSA), vancomycin-resistant enterococci (VRE), some gram-negative bacilli (e.g., *Acinetobacter baumannii*), and norovirus [1–6].

Personal protective equipment (PPE) is intended to reduce the risk that personnel will contaminate their skin and clothing during contact with patients and contaminated surfaces. However, such contamination is not uncommon despite use of PPE [7–10]. Failure to use PPE correctly is one factor that may result in contamination. For example, Landelle et al. found that 1 or more contacts with a patient or environmental surface in a *Clostridium difficile* infection (CDI) isolation room without wearing gloves was an independent risk factor for hand contamination with spores [9]. Failure to use PPE correctly may occur in part because personnel may not appreciate the risk for contamination after brief encounters or after contact only with environmental surfaces [11]. Incorrect donning and doffing technique is also common, and is associated with increased risk of contamination of skin and clothing [7, 12]. Thus, there is a need for improved strategies for training of personnel in correct use of PPE.

Several recent studies have demonstrated that simulations using fluorescent lotions or powders can be useful in understanding the spread of pathogens and in devising effective control strategies [7, 13–15]. For example, we showed that personnel frequently contaminated their skin and clothing during removal of fluorescent lotion-contaminated PPE and training that included use of the lotion to provide visual feedback reduced contamination [7]. The primary goal of the current study was to examine the use of fluorescent lotion as a means to track dissemination of pathogens to the environment and personnel during simulated patient care interactions. A secondary goal was to test whether an educational intervention would reduce the risk for contamination of the skin and clothing of personnel.

## Methods

We conducted a simulation study of pathogen dissemination and a quasi-experimental evaluation of the impact of an educational intervention. During simulations of patient care performed by 10 healthcare personnel, we evaluated environmental and personnel dissemination of fluorescent lotion and compared the frequency of skin and clothing contamination before versus after an educational intervention that included education and practice in PPE donning and doffing technique as described previously [7]. There was no concurrent control group that did not receive the educational intervention. A convenience sample of healthcare personnel providing direct care of patients in contact isolation participated in the study. Individual personnel participated in 3 simulations before the intervention and 3 simulations after the intervention during a 1 month period. Education included a 10-min video presentation and 20 min of practice in glove and gown donning and doffing. The donning and doffing protocols recommended by the Centers for Disease Control and Prevention (CDC) were presented [16]. Personnel practiced removal of fluorescent lotion-contaminated gloves and gown with use of a black light (Ultra Light UV1 by Grizzly Gear, SCS Direct Inc, Milford, CT) to identify sites of contamination. Personnel continued to practice until they were confident they could use the correct technique and avoid contamination.

The simulation room contained a life-size mannequin placed in a hospital bed in addition to a bedside table placed over the bed above the mannequin, call button placed on the bedside table, curtain, intravenous (IV) pole, stethoscope hung on the IV pole, and trashcan for disposal of PPE. For the assessments of the frequency of skin and environmental contamination, a solution containing 0.5 mL of phosphate-buffered saline containing 10^4^ plaque forming units (PFUs) of bacteriophage MS2 mixed with 0.5 mL of fluorescent lotion (Glitterbug Lotion, Brevis Corporation, Salt Lake City, UT) was applied to the mannequin’s anterior chest and abdomen, spread to cover a 10×10 cm area, and allowed to dry for 2 h. Bacteriophage MS2 is a non-pathogenic, non-enveloped RNA virus commonly used to study spread of pathogens [7]. The bacteriophage was prepared as previously described [7]. The contamination was applied to the mannequin because our primary goal was to simulate how contamination may spread from a patient to the environment and personnel during routine patient care activities.

For the simulations, healthcare personnel donned a cover gown (SafetyPlus Polyethylene Gown; TIDI Products, Neenah, WI) and nitrile gloves (Denville Scientific Inc, Metuchen, NJ). The clinical scenario involved the following steps: drawing the privacy curtain after entering the room, pressing the nurse call button to call for additional personnel, moving the bedside table away from the bed, raising the bed to the proper height for examination, taking the stethoscope from the IV pole, examining the mannequin by auscultating the chest and palpating the abdomen and returning everything in the room to its previous location, closing the privacy curtain, exiting the room, and removing PPE. The scenario was read to the participants prior to the simulations and they were cued if they forgot the sequence during the simulation. After each simulation, research personnel used a black light to assess for fluorescent lotion contamination of surfaces in the environment and of the skin and clothing of volunteers after PPE removal. The hands and wrists were then sampled for bacteriophage MS2 using a 4x4 gauze pad pre-moistened with phosphate-buffered saline. After every 10 simulations, the bedside table (top, short and long sides), side rail, stethoscope, IV pole, call button, curtain, trash can and floor were sampled for bacteriophage MS2 using pre-moistened BD BBL™ CultureSwabs™ (Becton Dickinson, Cockeysville, MD) and the surfaces were thoroughly cleaned and disinfected with bleach to eliminate fluorescent lotion and MS2. Sampling for MS2 on surfaces was performed after 10 simulations to determine accumulation after multiple repetitions. The swabs and gauze pads were cultured to quantify bacteriophage MS2 as previously described [7].

The percentage of contamination with fluorescent lotion was determined for each environmental site after each simulation; the average contamination of each site for the 3 repetitions of 10 simulations was graphed. The frequency of environmental surface and personnel skin and/or clothing contamination before versus after the intervention was compared using the Fischer’s exact test. Data were analyzed using R version 3.1.1.

## Results

**Fig. 1:**
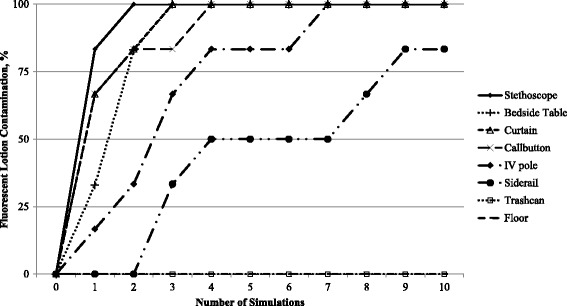
Progressive environmental surface contamination with fluorescent lotion during successive simulations of patient care

**Fig. 2:**
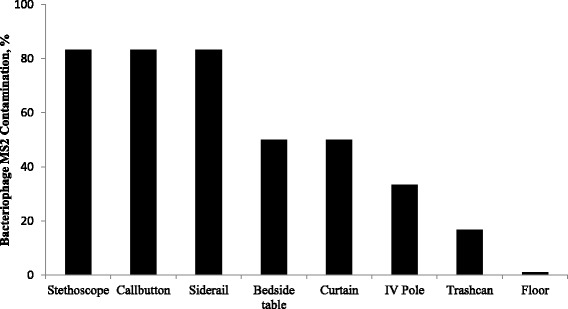
Overall distribution of environmental surface contamination with bacteriophage MS2

Ten healthcare personnel (5 physicians and 5 allied health providers) participated in 30 pre-intervention and 30 post-intervention simulations. The average age of the participants was 37 years (range, 24 to 55) and the average number of years working in healthcare was 8 (range, 3 to 26). Figure 1 shows the average accumulation of contamination with fluorescent lotion on surfaces during 6 sets of 10 simulations (i.e., surfaces were cleaned and disinfected after each set of 10 simulations). Multiple surfaces were contaminated after a single simulation and after 4 simulations 100 % of several surfaces were contaminated (stethoscopes, bedside table, curtain, and call button). No fluorescent lotion contamination of the trashcan or floor adjacent to the trashcan occurred during any of the simulations. As shown Fig. 2, the overall distribution of contamination with bacteriophage MS2 was similar to that of fluorescent lotion after 10 simulations.

**Fig. 3:**
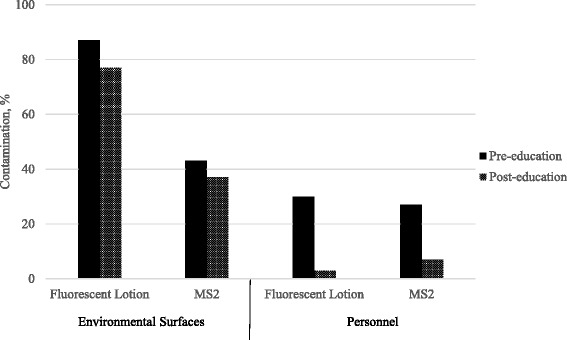
Contamination of environmental surfaces and healthcare personnel with fluorescent lotion and bacteriophage MS2 before and after an educational intervention

After the intervention, the overall percentages of environmental contamination with fluorescent lotion (26/30, 87 % versus 23/30, 77 %; *P* = 0.50) or bacteriophage MS2 (13/30, 43 % versus 12/30, 37 %; *P* = 1.00) were not significantly different than before the intervention (Fig. 3). However, skin and/or clothing contamination with fluorescent lotion decreased significantly after the intervention (9/30, 30 % versus 1/30, 3 %; *P* = 01). The percentage of skin and/or clothing contamination with bacteriophage MS2 also decreased after the intervention, but the reduction was not statistically significant (8/30, 27 % versus 2/30, 7 %; *P* = 0.08). However, there was a significant reduction in the concentration of bacteriophage MS2 recovered from hands after the intervention (0.31 versus 0.07 log_10_PFUs; *P* < 0.01). The most common skin site contaminated with fluorescent lotion was the dorsum of the wrist and the most common site of clothing contamination was the chest and abdomen.

## Discussion

During simulated patient care interactions, we found that fluorescent lotion was rapidly disseminated from a contaminated mannequin to touched surfaces throughout the room. The distribution of contamination by lotion was similar to the distribution of bacteriophage MS2. Our findings suggest that simulations with fluorescent lotion could be a useful teaching tool to illustrate the spread of pathogens. Such simulations could be useful for training of healthcare personnel. As noted previously, failure to use PPE correctly may occur in part due to lack of appreciation of the risk for contamination after brief contact or after contact with surfaces [11].

Our findings expand on previous studies that have used fluorescent tracers to assess contamination and train staff. We examined dissemination of fluorescent lotion from a contaminated mannequin to touched surfaces during simulated patient care interactions, whereas many previous studies have directly contaminated gowns and/or gloves and focused primarily on contamination of personnel [7, 10, 13]. Our results are consistent with a recent study in which transfer of fluorescent lotion from a contaminated mannequin to personnel and the environment occurred during simulated emergency department scenarios [17]. Our finding that a PPE training intervention that included practice with fluorescent lotions was effective in reducing skin and clothing contamination after PPE removal is consistent with a growing body of evidence that such interventions may be beneficial [7, 10, 13, 18, 19].

Although contamination of skin and clothing was reduced by the intervention, it was not reduced to zero. Additional measures such as disinfection of PPE prior to removal may be beneficial to further reduce contamination. Current guidelines from the CDC recommend that personnel disinfect their gloves at multiple steps during doffing of PPE used in the care of patients with suspected or confirmed Ebola virus infection [20]. However, this approach may not be practical for routine patient care. Improvements in PPE design are also needed to provide products that are easy to remove while minimizing the risk for self-contamination. For example, we demonstrated that a prototype seamless PPE design that ensures wrist coverage and requires the wearer to remove gloves and gowns simultaneously reduced hand and wrist contamination [21].

Our study has some limitations. Only a small cohort of subjects was studied and no nurses were included. However, we have previously reported that the frequency of contamination during removal of contaminated PPE is similar for nurses, physicians, and allied health personnel [7]. The frequency of contamination was relatively high in comparison to studies that have involved cultures during actual patient care [8, 9]. Thus, our results may mimic situations in which relatively heavy contamination is present. The post-intervention assessments were conducted within 1 month of the intervention. It is likely that intermittent training sessions will be necessary to maintain skills in PPE technique. Repeated training sessions would be particularly indicated during outbreaks of infection due to virulent pathogens. Finally, our conclusions are limited by the fact that we did not include a concurrent control group that did not receive the educational intervention. Studies with untrained control groups are needed for optimal evaluation of the efficacy of PPE training interventions.

## Conclusion

Fluorescent lotion was rapidly disseminated from a contaminated mannequin to touched surfaces throughout the room during simulations of patient care and the distribution of contamination was similar to the distribution of bacteriophage MS2. Our findings suggest that simulations with fluorescent lotion can be a useful teaching tool to illustrate the spread of pathogens and to train personnel in correct use of PPE.

## Abbreviations

CDC: Centers for disease control and prevention
CDI: *Clostridium difficile* infection
IV: Intravenous
MRSA: Methicillin-resistant *Staphylococcus aureus*
PFU: Plaque forming unit
PPE: Personal protective equipment
VRE: Vancomycin-resistant enterococci

## References

[1] Weber DJ, Anderson D, Rutala WA (2013). The role of the surface environment in healthcare-associated infections. Curr Opin Infect Dis.

[2] Hota B (2004). Contamination, disinfection, and cross-colonization: are hospital surfaces reservoirs for nosocomial infection?. Clin Infect Dis.

[3] Donskey CJ (2013). Does improving surface cleaning and disinfection reduce health care-associated infections?. Am J Infect Control.

[4] Hayden MK, Blom DW, Lyle EA, Moore CG, Weinstein RA (2008). Risk of hand or glove contamination after contact with patients colonized with vancomycin-resistant enterococcus or the colonized patients’ environment. Infect Control Hosp Epidemiol.

[5] Stiefel U, Cadnum JL, Eckstein BC, Guerrero DM, Tima MA, Donskey CJ (2011). Contamination of hands with methicillin-resistant *Staphylococcus aureus* after contact with environmental surfaces and after contact with the skin of colonized patients. Infect Control Hosp Epidemiol.

[6] Guerrero DM, Nerandzic MM, Jury LA, Jinno S, Chang S, Donskey CJ (2012). Acquisition of spores on gloved hands after contact with the skin of patients with *Clostridium difficile* infection and with environmental surfaces in their rooms. Am J Infect Control.

[7] Tomas ME, Kundrapu S, Thota P (2015). Contamination of the skin and clothing of healthcare personnel during removal of personal protective equipment. JAMA Intern Med.

[8] Morgan DJ, Rogawski E, Thom KA (2012). Transfer of multidrug-resistant bacteria to healthcare workers' gloves and gowns after patient contact increases with environmental contamination. Crit Care Med.

[9] Landelle C, Verachten M, Legrand P, Girou E, Barbut F, Brun-Buisson C (2014). Contamination of healthcare workers' hands with *Clostridium difficile* spores after caring for patients with *C. difficile* infection. Infect Control Hosp Epidemiol.

[10] Casanova L, Alfano-Sobsey E, Rutala WA, Weber DJ, Sobsey M (2008). Virus transfer from personal protective equipment to healthcare employees’ skin and clothing. Emerg Infect Dis.

[11] Dedrick RE, Sinkowitz-Cochran RL, Cunningham C (2007). Hand hygiene practices after brief encounters with patients: an important opportunity for prevention. Infect Control Hosp Epidemiol.

[12] Zellmer C, van Hoof S, Safdar N (2015). Variation in health care worker removal of personal protective equipment. Am J Infect Control.

[13] Cassir N, Boudjema S, Roux V, Reynier P, Brouqui P (2015). Infectious diseases of high consequence and personal protective equipment: a didactic method to assess the risk of contamination. Infect Control Hosp Epidemiol.

[14] Clay KA, O’Shea MK, Fletcher T (2015). Use of an ultraviolet tracer in simulation training for the clinical management of Ebola virus disease. J Hosp Infect.

[15] Oberyszyn AS, Robertson FM (2001). Novel rapid method for visualization of extent and location of aerosol contamination during high-speed sorting of potentially biohazardous samples. Cytometry.

[16] Centers for Disease Control and Prevention. Sequence for donning and removing personal protective equipment, 2014. www.cdc.gov/hai/pdfs/ppe/PPE-Sequence.pdf. Accessed 15 April 2016.

[17] Drew JL, Turner J, Mugele J (2016). Beating the spread: developing a simulation analog for contagious body fluids. Simul Healthc.

[18] Guo YP, Li Y, Wong PL (2014). Environment and body contamination: a comparison of two different removal methods in three types of personal protective clothing. Am J Infect Control.

[19] Lai JY, Guo YP, Or PP, Li Y (2011). Comparison of hand contamination rates and environmental contamination levels between two different glove removal methods and distances. Am J Infect Control.

[20] Centers for Disease Control and Prevention, 2014 Guidance on Personal Protective Equipment To Be Used by Healthcare Workers During Management of Patients with Ebola Virus Disease in U.S. Hospitals, Including Procedures for Putting On (Donning) and Removing (Doffing). http://www.cdc.gov/vhf/ebola/healthcare-us/ppe/guidance.html. Accessed 15 April 2016.

[21] Tomas ME, Cadnum JL, Mana TS, Jencson AL, Donskey CJ (2016). Seamless Suits: Reducing Personnel Contamination Through Improved Personal Protective Equipment Design. Infect Control Hosp Epidemiol.

